# Supplementary material for: Supramolecularly directed rotary motion in a photoresponsive receptor
Source: Nat Commun. 2018 May 18;9:1984. doi: 10.1038/s41467-018-04249-x (PMC5959844; doi:10.1038/s41467-018-04249-x)
Supplement: Supplementary file 1 — Supplementary Information [file 41467_2018_4249_MOESM1_ESM.pdf]

Supramolecularly directed rotary motion in a photoresponsive receptor

Wezenberg et al.

Stratingh Institute for Chemistry, University of Groningen, Nijenborgh 4, 9747 AG, Groningen, The Netherlands

Email: s.j.wezenberg@rug.nl

Table of contents:

Supplementary Methods S2

VT NMR and NMR dilution studies S3

Chirality induction experiments S5

DFT Calculations S7

PSS determination by ^{1}H NMR S19

Quantum yield determination S21

^{1}H, ^{13}C and ^{31}P NMR Spectra S24

Supplementary References S32

Supplementary Methods

General methods and materials:

THF and CH_2Cl_2 were dried by using an MBraun solvent purification system. Anhydrous MeOH and pyridine were purchased from Acros Organics. The degassing of solvents was carried out by purging with Argon for at least 30 min. Tetrabutylammonium hydroxide-30 hydrate and phosphoryl chloride were purchased from Sigma-Aldrich and the enantiomers (*S*)- and (*R*)- 5,5’,6,6’,7,7’,8,8’-octahydro-1,1’-bi-2-naphthol were obtained from TCI. The isomers (*E*)- and (*Z*)-2,2',3,3'-tetrahydro-(1,1'-biindenylidene)-6,6'-diamine were prepared according to a procedure described in the literature.[1] All other chemicals were commercial products and were used as received. Flash chromatography (FC) was performed using silica gel (SiO_2) purchased from Machery-Nagel (0.04-0.063 mm) and thin-layer chromatography (TLC) was carried out on aluminium sheets coated with silica 60 F254 obtained from Merck; compounds were visualized with a UV lamp (254 nm). Melting points (m.p.) were determined using a Büchi-B545 capillary melting point apparatus. ^1H, ^{13}C and ^{31}P NMR spectra were recorded on Varian Mercury Plus-400, Agilent 400-MR, Varian Unity Plus-500 and Varian Innova-600 spectrometers at 298K unless indicated otherwise. Chemical shifts (δ) are denoted in parts per million (ppm) relative to CD_2Cl_2 (for ^1H detection, δ = 5.32 ppm; for ^{13}C detection, δ = 53.84 ppm), DMSO-d_6 (for ^1H detection, δ = 2.50 ppm; for ^{13}C detection, δ = 39.52 ppm) or aqueous 85% H_3PO_4 (for 31P detection, δ = 0.00 ppm). The splitting pattern of peaks is designated as follows: s (singlet), d (doublet), t (triplet), (multiplet), br (broad), or dd (doublet of doublets). High-resolution mass spectrometry (ESI-MS) was performed on a LTQ Orbitrap XL spectrometer with ESI ionisation. UV-vis spectra were recorded on a HP 8543 diode array spectrophotometer, equipped with a Quantum Northwest Peltier controller, in a 1 cm or 1 mm quartz cuvette. CD spectra were measured on a Jasco 810-CD instrument.

VT NMR and NMR dilution studies

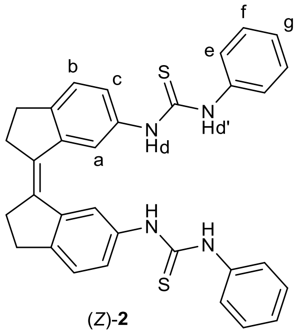

(Z)-**2**

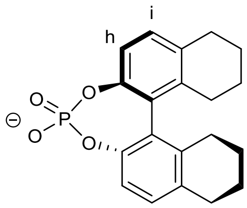

(S)-**3**

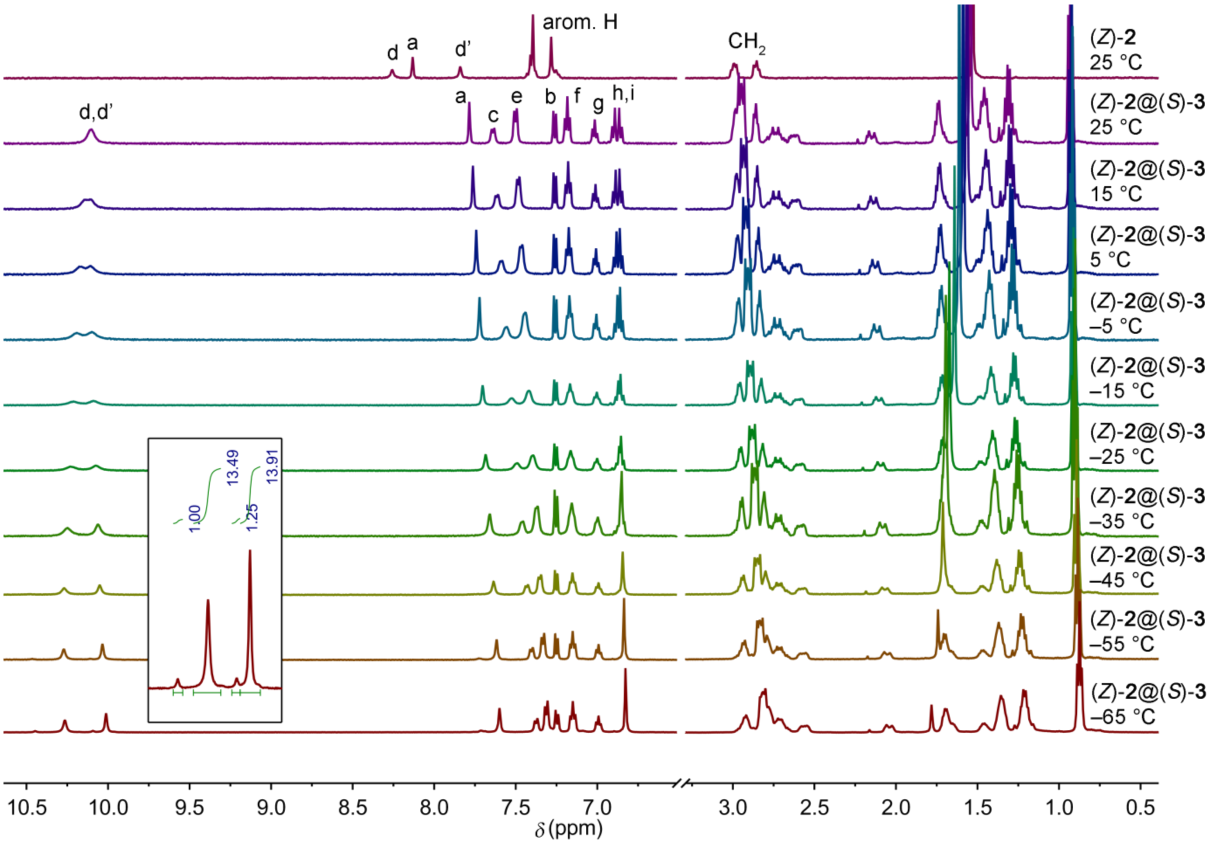

Supplementary Figure 1. Aromatic and aliphatic region in the ^{1}H NMR spectrum of (*Z*)-**2** (2 mM in CD_2Cl_2) before (top, 400 MHz) and after addition of 1 equivalent of $[Bu_4N]^{+}[(S)\text{-}\mathbf{3})]^{-}$ (below, 500 MHz). The ^{1}H NMR spectrum of the mixture was recorded at various temperatures ranging from 25 °C to –65 °C. Assignments are based on COSY and NOESY spectra.

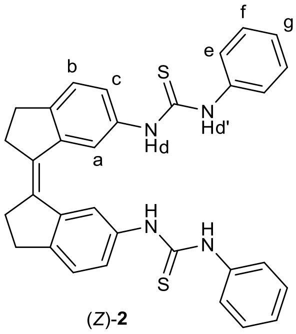

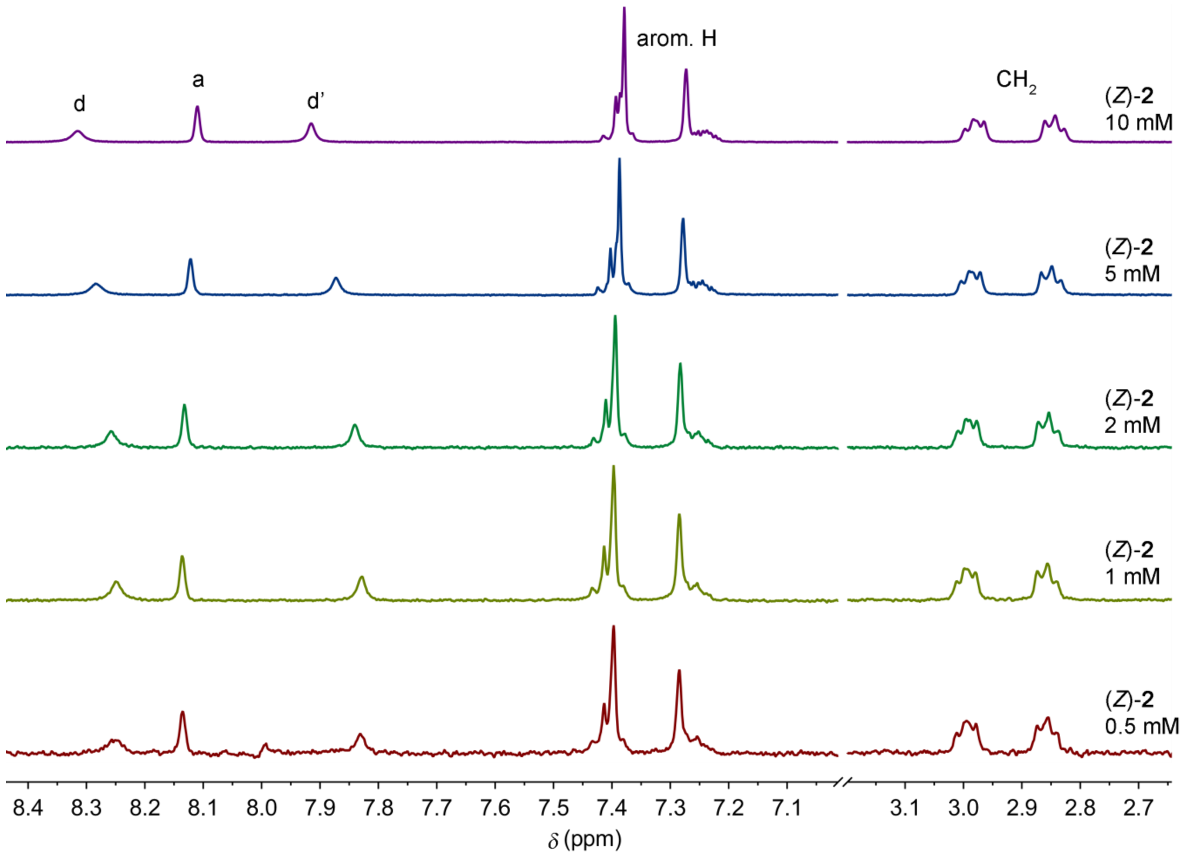

Supplementary Figure 2. Aromatic and aliphatic region in the ^1H NMR spectrum (400 MHz) of (*Z*)-**2** in CD_2Cl_2 at different concentrations ranging from 10 to 0.5 mM.

Chirality induction experiments

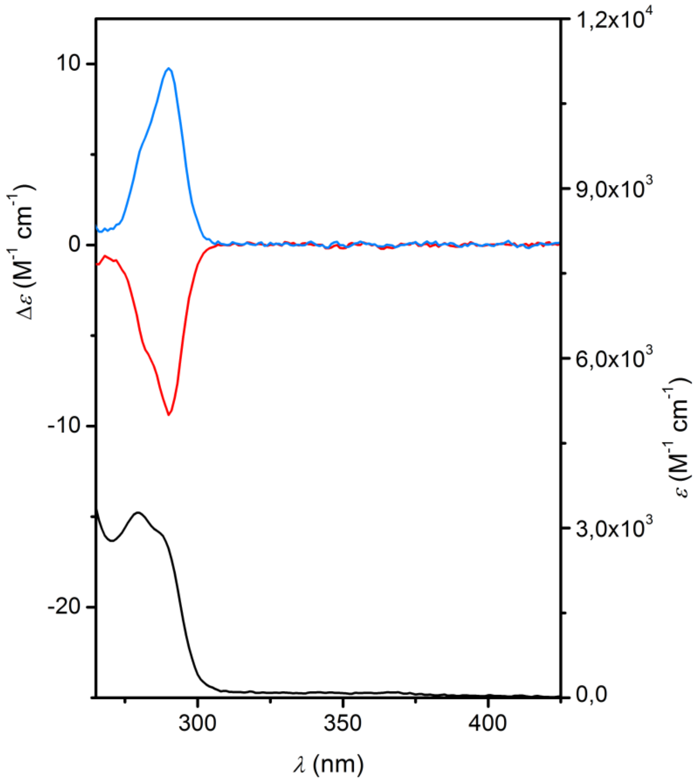

Supplementary Figure 3. CD (top) and UV-vis (bottom) absorption spectra of $[Bu_4N]^+[(S)$-**3**$)]^-$ (CD; red line) and $[Bu_4N]^+[(R)$-**3**$)]^-$ (CD; blue line) in CH_2Cl_2 ($c = 5.0 \times 10^{-4}$ M, 25 °C, 1 mm quartz cuvette).

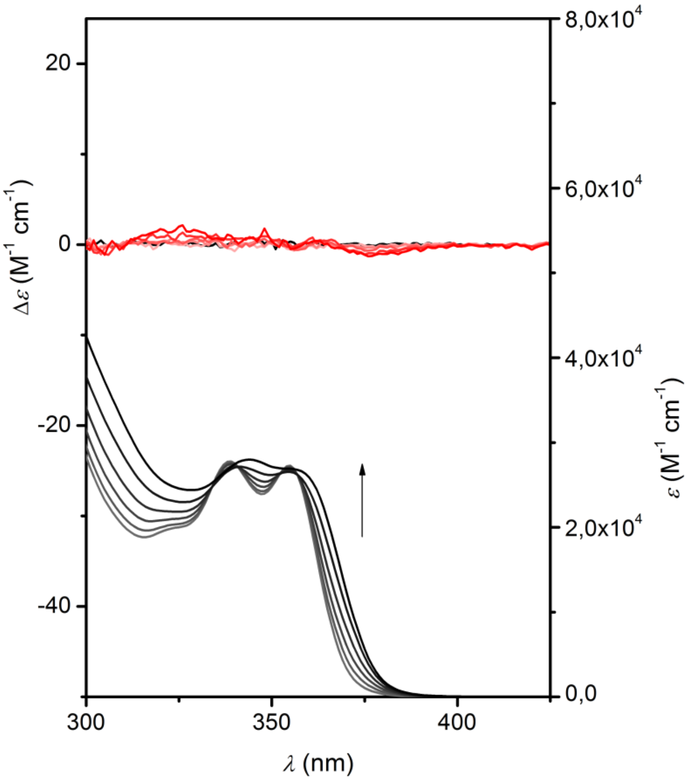

Supplementary Figure 4. CD (top) and UV-vis (bottom) spectral changes upon stepwise addition of $[Bu_4N]^+[(S)\text{-}\mathbf{3})]^-$ (0.0, 0.2, 0.5, 1.0, 2.0 and 4.0 equiv.) to (*E*)-**2** in CH_2Cl_2 ($c = 5.0 \times 10^{-4}$ M, 25 °C, 1 mm quartz cuvette).

DFT Calculations

The Gaussian 09 program[2] was used for geometry optimisations, calculation of energies and time-dependent DFT calculations. Initially different input geometries were optimised at the semi-empirical PM6 level to find the global minima.

Geometry optimisation and energy barrier for helicity inversion of stiff-stilbene. The geometries of the (*E*)-, (*P*)-(*Z*)- and (*M*)-(*Z*)-isomer (Supplementary Fig. 5) were optimised first at the PM6 level and a transition state was found by a central dihedral scan. Additional optimisation and frequency analysis were performed on the DFT B3LYP/6-31G+(d,p) level of theory using tight convergence criteria. The DFT-optimized structures were found to have zero imaginary frequencies, except for the transition state, which had one imaginary frequency; $\Delta^{\ddagger}G_{298}$ = 16.7 kJ mol^{-1}. Cartesian coordinates are given in Supplementary Table 1-4.

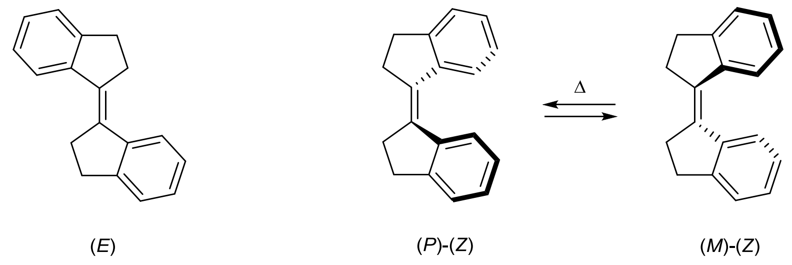

Supplementary Figure 5. Line drawings of the (*E*)- and (*Z*)-isomer of stiff-stilbene, showing the possible (*P*)- and (*M*)-helical conformations for the latter.

Geometry optimisation of (*P*)-(*Z*)-2@(*S*)-3 and (*M*)-(*Z*)-2@(*S*)-3. Two conformations of the octahydro-1,1'-bi-2-naphthol unit, both found in reported solid state structures,[3] were considered. In one conformation the hydrogen atoms in the 8,8'-positions point toward each other (H_{in}), while in the other they point away from each other (H_{out}, see Supplementary Fig. 6). Structures with H_{in} were found to be the lowest in energy on the PM6 level. Further geometry optimisation, frequency analysis and time-dependent DFT calculations were performed on the DFT B3LYP/6-31G++(d,p) level of theory using tight convergence criteria and an IEFPCM CH_2Cl_2 solvation model. The DFT-optimized structures were found to have zero imaginary frequencies. Cartesian coordinates are given in Supplementary Table 5-6.

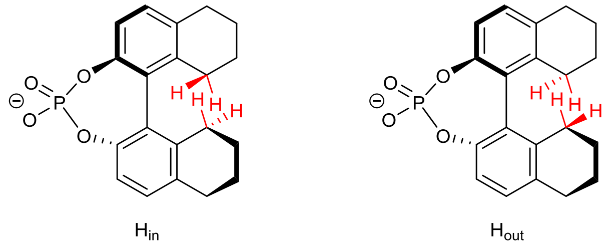

Supplementary Figure 6. Possible conformations of (*S*)-5,5’,6,6’,7,7’,8,8’-octahydro-1,10-binaphthyl-2,2’-diyl phosphate considered in the DFT geometry optimisations.

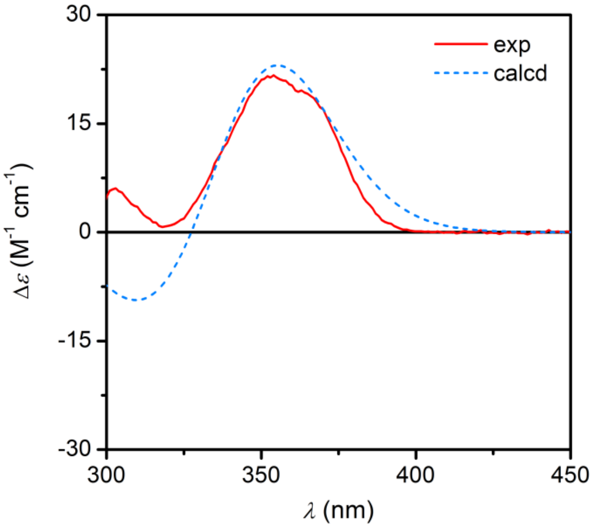

Supplementary Figure 7. Experimental (red, solid) and calculated (blue, dashed) CD spectra of (*P*)-(*Z*)-**2**@(*S*)-**3** (30 electronic transitions were computed). Intensities were normalised and the calculated spectrum was blue-shifted by 18 nm for easier comparison.

Supplementary Table 1. Cartesian coordinates of (*E*)-stiff-stilbene.

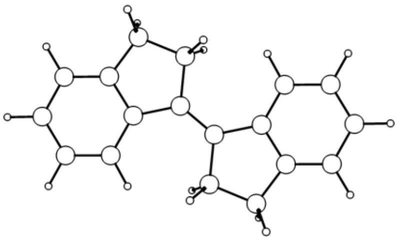

symmetry c1

atom	X	Y	Z
C	0.574619000	-0.364439000	-0.000002000
C	-0.574617000	0.364425000	0.000001000
C	-1.965646000	-0.128347000	0.000003000
C	-2.866186000	0.960645000	0.000007000
C	-2.135415000	2.280672000	0.000010000
C	-0.635284000	1.892301000	0.000003000
C	1.965643000	0.128341000	-0.000004000
C	2.866191000	-0.960644000	-0.000005000
C	2.135428000	-2.280676000	-0.000006000
C	0.635295000	-1.892314000	-0.000003000
C	-2.490905000	-1.435629000	0.000001000
C	-3.874220000	-1.630044000	0.000002000
C	-4.752718000	-0.541327000	0.000006000
C	-4.244263000	0.761732000	0.000009000
C	2.490892000	1.435627000	-0.000005000
C	3.874205000	1.630054000	-0.000006000
C	4.752711000	0.541343000	-0.000007000
C	4.244267000	-0.761720000	-0.000006000
H	-5.826148000	-0.707521000	0.000007000
H	5.826140000	0.707545000	-0.000008000
H	-2.400144000	2.881447000	0.878575000
H	-2.400152000	2.881456000	-0.878546000
H	-0.124414000	2.301987000	-0.878872000
H	2.400167000	-2.881455000	0.878553000
H	2.400164000	-2.881452000	-0.878568000
H	0.124422000	-2.302003000	-0.878874000
H	-0.124405000	2.301986000	0.878872000
H	0.124425000	-2.302004000	0.878870000
H	-1.846162000	-2.303218000	-0.000002000
H	-4.920665000	1.613204000	0.000012000
H	1.846140000	2.303211000	-0.000004000
H	4.920675000	-1.613188000	-0.000007000
H	-4.269138000	-2.642151000	0.000001000
H	4.269115000	2.642164000	-0.000007000

Sum of electronic and thermal Free Energies = –695.358498

Supplementary Table 2. Cartesian coordinates of (*P*)-(*Z*)-stiff-stilbene.

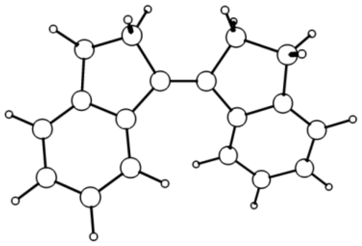

symmetry c1

atom	X	Y	Z
C	0.678285000	0.997951000	-0.008251000
C	-0.678285000	0.997951000	0.008251000
C	-1.664245000	-0.097786000	0.109983000
C	-2.944031000	0.382585000	-0.243682000
C	-2.873391000	1.838982000	-0.642097000
C	-1.495202000	2.287137000	-0.093985000
C	1.664245000	-0.097786000	-0.109983000
C	2.944031000	0.382585000	0.243682000
C	2.873391000	1.838982000	0.642097000
C	1.495202000	2.287137000	0.093985000
C	-1.535030000	-1.411242000	0.589009000
C	-2.661981000	-2.233152000	0.661419000
C	-3.919895000	-1.760921000	0.266472000
C	-4.064345000	-0.442820000	-0.180511000
C	1.535030000	-1.411242000	-0.589009000
C	2.661981000	-2.233152000	-0.661419000
C	3.919895000	-1.760921000	-0.266472000
C	4.064345000	-0.442820000	0.180511000
H	-4.787183000	-2.412196000	0.327083000
H	4.787183000	-2.412196000	-0.327083000
H	-3.705936000	2.428591000	-0.244467000
H	-2.900668000	1.939974000	-1.736079000
H	-1.016708000	3.040204000	-0.726181000
H	3.705936000	2.428591000	0.244467000
H	2.900668000	1.939974000	1.736079000
H	1.016708000	3.040204000	0.726181000
H	-1.623028000	2.733641000	0.903163000
H	1.623028000	2.733641000	-0.903163000
H	-0.577385000	-1.784469000	0.932192000
H	-5.044215000	-0.063613000	-0.460364000
H	0.577385000	-1.784469000	-0.932192000
H	5.044215000	-0.063613000	0.460364000
H	-2.561314000	-3.246533000	1.040028000
H	2.561314000	-3.246533000	-1.040028000

Sum of electronic and thermal Free Energies = –695.356049

Supplementary Table 3. Cartesian coordinates of (*M*)-(*Z*)-stiff-stilbene.

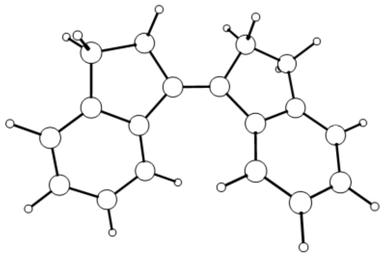

symmetry c1

atom	X	Y	Z
C	0.678285000	0.997951000	-0.008251000
C	-0.678285000	0.997951000	0.008251000
C	-1.664245000	-0.097786000	0.109983000
C	-2.944031000	0.382585000	-0.243682000
C	-2.873391000	1.838982000	-0.642097000
C	-1.495202000	2.287137000	-0.093985000
C	1.664245000	-0.097786000	-0.109983000
C	2.944031000	0.382585000	0.243682000
C	2.873391000	1.838982000	0.642097000
C	1.495202000	2.287137000	0.093985000
C	-1.535030000	-1.411242000	0.589009000
C	-2.661981000	-2.233152000	0.661419000
C	-3.919895000	-1.760921000	0.266472000
C	-4.064345000	-0.442820000	-0.180511000
C	1.535030000	-1.411242000	-0.589009000
C	2.661981000	-2.233152000	-0.661419000
C	3.919895000	-1.760921000	-0.266472000
C	4.064345000	-0.442820000	0.180511000
H	-4.787183000	-2.412196000	0.327083000
H	4.787183000	-2.412196000	-0.327083000
H	-3.705936000	2.428591000	-0.244467000
H	-2.900668000	1.939974000	-1.736079000
H	-1.016708000	3.040204000	-0.726181000
H	3.705936000	2.428591000	0.244467000
H	2.900668000	1.939974000	1.736079000
H	1.016708000	3.040204000	0.726181000
H	-1.623028000	2.733641000	0.903163000
H	1.623028000	2.733641000	-0.903163000
H	-0.577385000	-1.784469000	0.932192000
H	-5.044215000	-0.063613000	-0.460364000
H	0.577385000	-1.784469000	-0.932192000
H	5.044215000	-0.063613000	0.460364000
H	-2.561314000	-3.246533000	1.040028000
H	2.561314000	-3.246533000	-1.040028000

Sum of electronic and thermal Free Energies = –695.356049

Supplementary Table 4. Cartesian coordinates of transition state.

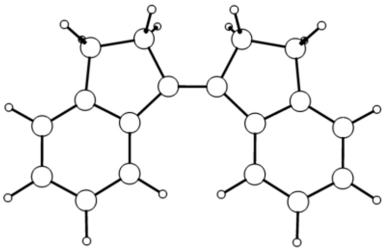

symmetry c1

atom	X	Y	Z
C	0.678285000	0.997951000	-0.008251000
C	-0.678285000	0.997951000	0.008251000
C	-1.664245000	-0.097786000	0.109983000
C	-2.944031000	0.382585000	-0.243682000
C	-2.873391000	1.838982000	-0.642097000
C	-1.495202000	2.287137000	-0.093985000
C	1.664245000	-0.097786000	-0.109983000
C	2.944031000	0.382585000	0.243682000
C	2.873391000	1.838982000	0.642097000
C	1.495202000	2.287137000	0.093985000
C	-1.535030000	-1.411242000	0.589009000
C	-2.661981000	-2.233152000	0.661419000
C	-3.919895000	-1.760921000	0.266472000
C	-4.064345000	-0.442820000	-0.180511000
C	1.535030000	-1.411242000	-0.589009000
C	2.661981000	-2.233152000	-0.661419000
C	3.919895000	-1.760921000	-0.266472000
C	4.064345000	-0.442820000	0.180511000
H	-4.787183000	-2.412196000	0.327083000
H	4.787183000	-2.412196000	-0.327083000
H	-3.705936000	2.428591000	-0.244467000
H	-2.900668000	1.939974000	-1.736079000
H	-1.016708000	3.040204000	-0.726181000
H	3.705936000	2.428591000	0.244467000
H	2.900668000	1.939974000	1.736079000
H	1.016708000	3.040204000	0.726181000
H	-1.623028000	2.733641000	0.903163000
H	1.623028000	2.733641000	-0.903163000
H	-0.577385000	-1.784469000	0.932192000
H	-5.044215000	-0.063613000	-0.460364000
H	0.577385000	-1.784469000	-0.932192000
H	5.044215000	-0.063613000	0.460364000
H	-2.561314000	-3.246533000	1.040028000
H	2.561314000	-3.246533000	-1.040028000

Sum of electronic and thermal Free Energies = –695.349700

Supplementary Table 5. Cartesian coordinates of (*P*)-(*Z*)-**2**@(*S*)-**3**.

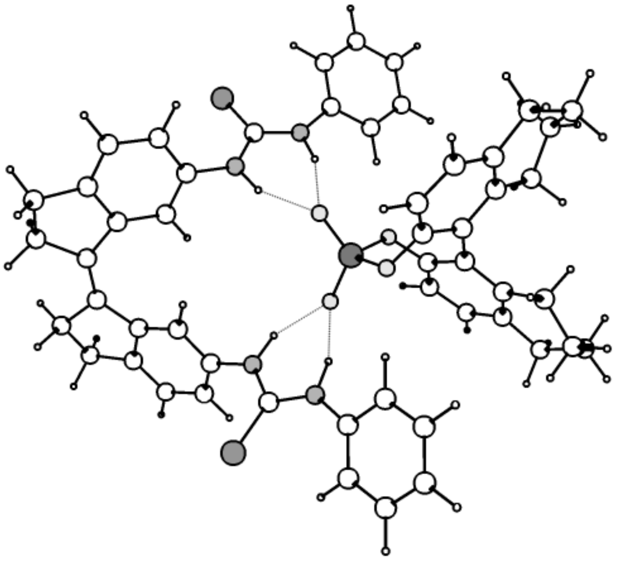

symmetry c1

atom	X	Y	Z
C	-6.837227000	0.558667000	0.385340000
C	-6.837227000	-0.558692000	-0.385319000
C	-5.751808000	-1.441215000	-0.861956000
C	-6.249052000	-2.294883000	-1.869987000
C	-7.697964000	-1.989181000	-2.164936000
C	-8.134570000	-1.155435000	-0.935695000
C	-5.751811000	1.441196000	0.861969000
C	-6.249054000	2.294863000	1.870002000
C	-7.697962000	1.989154000	2.164961000
C	-8.134570000	1.155403000	0.935723000
C	-4.445912000	-1.633660000	-0.379848000
C	-3.650402000	-2.644181000	-0.929665000
C	-4.135129000	-3.447702000	-1.972880000
C	-5.441119000	-3.279713000	-2.435516000
C	-4.445918000	1.633647000	0.379855000
C	-3.650411000	2.644172000	0.929666000
C	-4.135136000	3.447693000	1.972881000
C	-5.441123000	3.279699000	2.435525000
C	-1.817660000	3.945604000	-0.138990000
N	-0.470005000	3.859600000	-0.344580000
S	-2.800860000	5.256804000	-0.601602000
C	0.437528000	4.744960000	-0.970973000
C	0.339490000	6.144456000	-0.916164000
C	1.319249000	6.933779000	-1.524756000
C	2.409770000	6.352367000	-2.178088000
C	2.518046000	4.958198000	-2.211795000

C	1.542608000	4.158831000	-1.615331000
C	-1.817639000	-3.945607000	0.138975000
N	-0.469984000	-3.859595000	0.344562000
S	-2.800828000	-5.256816000	0.601583000
C	0.437554000	-4.744952000	0.970951000
C	0.339530000	-6.144448000	0.916126000
C	1.319293000	-6.933769000	1.524714000
C	2.409806000	-6.352354000	2.178057000
C	2.518069000	-4.958185000	2.211780000
C	1.542626000	-4.158820000	1.615320000
N	-2.318262000	2.822199000	0.451270000
N	-2.318251000	-2.822202000	-0.451277000
P	0.782375000	0.000007000	0.000001000
O	1.835368000	0.629944000	-1.113688000
O	0.035934000	1.188144000	0.557915000
H	-3.493472000	-4.213399000	-2.394447000
H	-3.493481000	4.213394000	2.394443000
H	-7.787319000	-1.392656000	-3.083387000
H	-8.300049000	-2.891938000	-2.307844000
H	-8.595158000	-1.815344000	-0.186205000
H	-7.787308000	1.392629000	3.083413000
H	-8.300051000	2.891908000	2.307870000
H	-8.595167000	1.815308000	0.186235000
H	-0.063769000	2.949595000	-0.097875000
H	-0.496264000	6.605431000	-0.408918000
H	1.227758000	8.015397000	-1.477045000
H	3.165818000	6.974664000	-2.647673000
H	3.361710000	4.486420000	-2.707468000
H	1.633392000	3.076471000	-1.640166000
H	-0.063755000	-2.949586000	0.097863000
H	-0.496217000	-6.605426000	0.408870000
H	1.227812000	-8.015388000	1.476990000
H	3.165857000	-6.974649000	2.647639000
H	3.361726000	-4.486404000	2.707462000
H	1.633399000	-3.076460000	1.640168000
H	-1.673406000	2.033291000	0.558821000
H	-1.673401000	-2.033288000	-0.558823000
H	-8.873022000	-0.390066000	-1.188958000
H	-8.873016000	0.390030000	1.188992000
H	-4.054957000	-1.048316000	0.444459000
H	-5.825078000	-3.924404000	-3.221837000
H	-4.054965000	1.048304000	-0.444454000
H	-5.825081000	3.924389000	3.221847000
O	1.835364000	-0.629931000	1.113692000
C	4.958413000	1.879375000	2.310725000
C	4.995504000	1.290249000	1.029852000
C	3.910288000	1.562014000	3.188269000
C	2.866774000	0.730066000	2.798421000

C	3.987943000	0.360459000	0.656379000
C	3.987943000	-0.360449000	-0.656370000
C	2.905513000	0.163714000	1.527247000
C	2.905516000	-0.163702000	-1.527241000
C	6.061132000	1.714634000	0.027982000
C	2.866779000	-0.730054000	-2.798415000
C	4.995504000	-1.290240000	-1.029841000
C	6.061129000	-1.714628000	-0.027969000
C	6.714775000	-3.058501000	-0.372250000
C	5.986940000	-2.906357000	-2.765503000
C	4.958415000	-1.879367000	-2.310714000
C	7.189939000	3.046640000	1.827098000
C	3.910292000	-1.562004000	-3.188261000
C	5.986938000	2.906364000	2.765516000
C	7.189939000	-3.046635000	-1.827083000
C	6.714779000	3.058506000	0.372264000
O	0.035934000	-1.188128000	-0.557915000
H	3.892359000	2.012826000	4.177764000
H	5.620004000	1.748294000	-0.973848000
H	6.855780000	0.959979000	-0.018892000
H	5.619999000	-1.748287000	0.973861000
H	6.855778000	-0.959973000	0.018907000
H	5.996760000	-3.877223000	-0.226571000
H	7.550718000	-3.240876000	0.312816000
H	6.319243000	-2.657094000	-3.780934000
H	5.483955000	-3.880880000	-2.846017000
H	7.881952000	2.205287000	1.972378000
H	7.745107000	3.959702000	2.070693000
H	3.892365000	-2.012816000	-4.177756000
H	6.319239000	2.657101000	3.780947000
H	5.483954000	3.880888000	2.846028000
H	7.881953000	-2.205283000	-1.972361000
H	7.745105000	-3.959697000	-2.070677000
H	5.996766000	3.877230000	0.226584000
H	7.550723000	3.240880000	-0.312800000
H	2.021909000	0.529744000	3.448919000
H	2.021915000	-0.529731000	-3.448914000

Sum of electronic and thermal Free Energies = –3667.798708

Supplementary Table 6. Cartesian coordinates of (*M*)-(*Z*)-**2**@(*S*)-**3**.

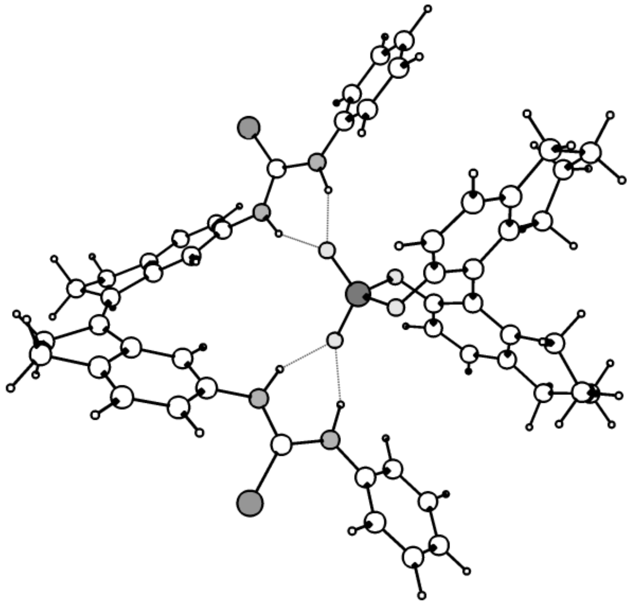

symmetry c1

atom	X	Y	Z
C	-6.648594000	0.459387000	-0.498739000
C	-6.648604000	-0.459340000	0.498694000
C	-5.553333000	-1.218530000	1.136239000
C	-6.020472000	-1.800876000	2.333011000
C	-7.458382000	-1.411735000	2.586998000
C	-7.930794000	-0.914676000	1.196470000
C	-5.553306000	1.218563000	-1.136273000
C	-6.020427000	1.800915000	-2.333049000
C	-7.458340000	1.411794000	-2.587049000
C	-7.930771000	0.914740000	-1.196525000
C	-4.277739000	-1.551693000	0.654288000
C	-3.472661000	-2.436000000	1.383881000
C	-3.915865000	-2.947495000	2.614644000
C	-5.195652000	-2.638548000	3.081072000
C	-4.277713000	1.551709000	-0.654310000
C	-3.472618000	2.436007000	-1.383895000
C	-3.915803000	2.947508000	-2.614662000
C	-5.195590000	2.638577000	-3.081102000
C	-1.659779000	4.009914000	-0.676920000
N	-0.399984000	3.953833000	-0.152610000
S	-2.499641000	5.455064000	-0.994632000
C	0.473463000	5.009166000	0.205724000
C	1.197589000	4.871908000	1.400692000
C	2.126941000	5.842910000	1.779389000
C	2.341764000	6.965171000	0.974174000

C	1.627498000	7.096248000	-0.221691000
C	0.704165000	6.124680000	-0.613808000
C	-1.659821000	-4.009925000	0.676941000
N	-0.400017000	-3.953852000	0.152656000
S	-2.499697000	-5.455068000	0.994651000
C	0.473437000	-5.009185000	-0.205660000
C	1.197612000	-4.871906000	-1.400597000
C	2.126972000	-5.842906000	-1.779278000
C	2.341756000	-6.965186000	-0.974079000
C	1.627443000	-7.096282000	0.221756000
C	0.704101000	-6.124717000	0.613857000
N	-2.188207000	2.764310000	-0.869925000
N	-2.188250000	-2.764317000	0.869923000
O	-0.187183000	-1.197295000	-0.521333000
O	1.622708000	0.601438000	-1.129900000
O	1.622716000	-0.601477000	1.129879000
C	4.761320000	-1.912282000	-2.276227000
C	4.788981000	-1.306368000	-1.003283000
C	3.717172000	-1.609389000	-3.163684000
C	2.667508000	-0.777937000	-2.789591000
C	3.777553000	-0.373089000	-0.648990000
C	3.777550000	0.373073000	0.648968000
C	2.696688000	-0.195064000	-1.525437000
C	2.696688000	0.195035000	1.525417000
C	5.847796000	-1.715597000	0.012203000
C	2.667503000	0.777905000	2.789572000
C	4.788968000	1.306364000	1.003261000
C	5.847775000	1.715609000	-0.012226000
C	6.508352000	3.061092000	0.311516000
C	5.796016000	2.941453000	2.711284000
C	4.761301000	1.912276000	2.276206000
C	6.993237000	-3.065410000	-1.763227000
C	3.717158000	1.609369000	3.163664000
C	5.796048000	-2.941446000	-2.711306000
C	6.993200000	3.065439000	1.763201000
C	6.508395000	-3.061070000	-0.311541000
O	-0.187187000	1.197246000	0.521319000
P	0.573873000	-0.000022000	-0.000008000
H	-3.269703000	-3.605620000	3.181992000
H	-3.269628000	3.605626000	-3.182003000
H	-7.516854000	-0.597036000	3.322189000
H	-8.059885000	-2.239979000	2.974280000
H	-8.389592000	-1.748564000	0.645418000
H	-7.516816000	0.597097000	-3.322241000
H	-8.059828000	2.240046000	-2.974336000
H	-8.389563000	1.748634000	-0.645477000
H	-0.093411000	3.018721000	0.142085000
H	1.028462000	4.002496000	2.029157000

H	2.677556000	5.720863000	2.707633000
H	3.059595000	7.724360000	1.270393000
H	1.795366000	7.956583000	-0.863488000
H	0.164595000	6.227739000	-1.545905000
H	-0.093438000	-3.018741000	-0.142039000
H	1.028516000	-4.002480000	-2.029051000
H	2.677624000	-5.720843000	-2.707498000
H	3.059593000	-7.724374000	-1.270285000
H	1.795281000	-7.956632000	0.863542000
H	0.164496000	-6.227791000	1.545932000
H	-1.652258000	1.992173000	-0.460378000
H	-1.652295000	-1.992186000	0.460373000
H	-8.677359000	-0.118713000	1.263021000
H	-8.677346000	0.118787000	-1.263084000
H	-3.923571000	-1.176562000	-0.300042000
H	-5.546118000	-3.068886000	4.015695000
H	-3.923559000	1.176572000	0.300023000
H	-5.546042000	3.068921000	-4.015728000
H	3.707458000	-2.071323000	-4.148237000
H	5.397489000	-1.740526000	1.010181000
H	6.639246000	-0.957628000	0.058597000
H	5.397467000	1.740530000	-1.010204000
H	6.639237000	0.957653000	-0.058621000
H	5.792081000	3.880214000	0.160206000
H	7.340315000	3.232143000	-0.381261000
H	6.134114000	2.704010000	3.727690000
H	5.296774000	3.918666000	2.782158000
H	7.682911000	-2.223211000	-1.914558000
H	7.553608000	-3.979200000	-1.991904000
H	3.707440000	2.071301000	4.148218000
H	6.134137000	-2.704003000	-3.727714000
H	5.296820000	-3.918666000	-2.782173000
H	7.682888000	2.223251000	1.914527000
H	7.553555000	3.979238000	1.991877000
H	5.792138000	-3.880204000	-0.160228000
H	7.340363000	-3.232106000	0.381233000
H	1.826063000	-0.588170000	-3.447683000
H	1.826062000	0.588128000	3.447665000

Sum of electronic and thermal Free Energies = – 3667.796589

PSS determination by ^{1}H NMR

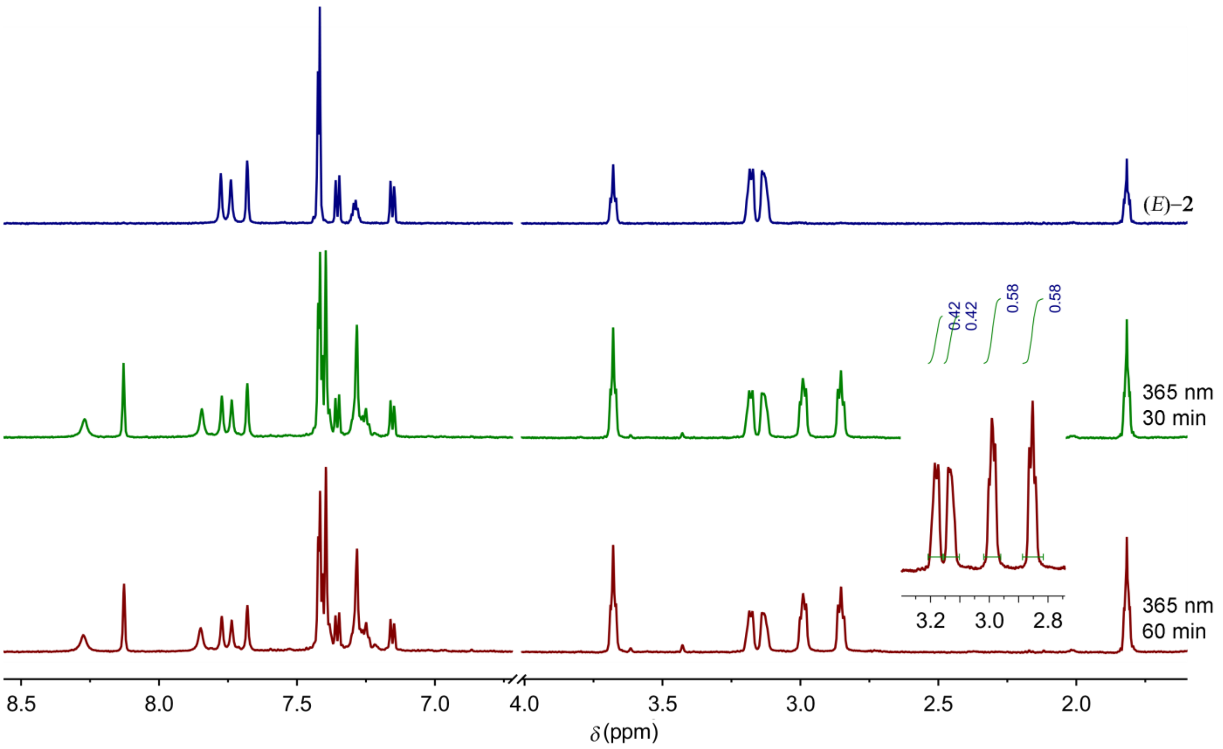

Supplementary Figure 8. (top) Aromatic and aliphatic region in the ^{1}H NMR spectrum (600 MHz) of (*E*)-**2** (2 mM) in degassed CD_2Cl_2. (below) Spectral changes upon irradiation with 365 nm light. The PSS *E*/*Z* ratio (42:58) was determined by integration of the CH_2 signals.

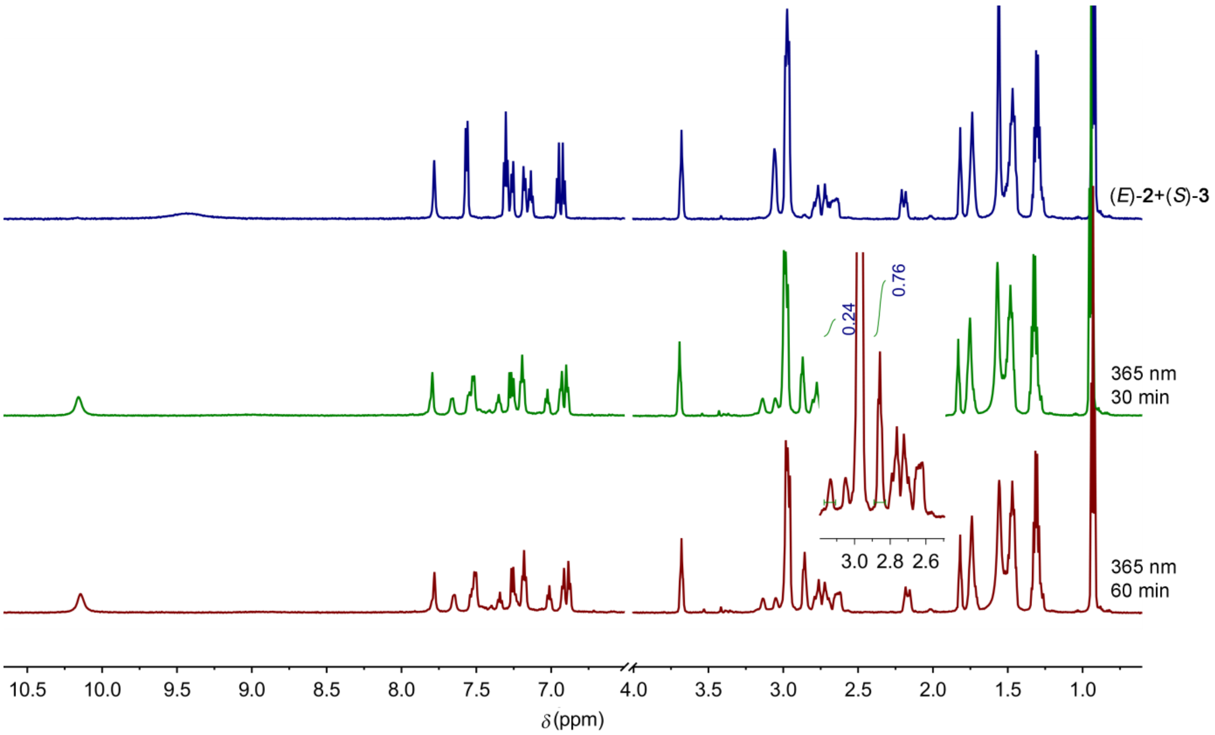

Supplementary Figure 9. (top) Aromatic and aliphatic region in the ^{1}H NMR spectrum (600 MHz) of a 1:1 mixture of (*E*)-**2** and $[NBu_4]^+$[(*S*)-**3**]$^-$ (2 mM) in degassed CD_2Cl_2. (below) Spectral changes upon irradiation with 365 nm light. The PSS *E*/*Z* ratio (24:76) was determined by integration of the CH_2 signals.

Quantum yield determination

An aqueous 0.05 M H_2SO_4 solution containing 6 mM potassium ferrioxalate (2 mL, 1 cm quartz cuvette) was irradiated at 20 °C for 30, 60, 90, 120 or 150 s in the dark with the Thorlab model F365F1 high-power LED (λ_{max} = 365 nm). A volume of 1 mL was taken and diluted 3× with an aqueous 0.5 M H_2SO_4 solution containing phenanthroline (0.1 wt%). The absorption at λ = 510 nm was measured and compared to an identically prepared non-irradiated sample. The concentration of $[Fe(phenanthroline)_3]^{2+}$ complex was calculated using its molar absorptivity (ε = 11100 m^{-1} cm^{-1}). This concentration corresponded to ⅓ (cf. 3× diluted) of the concentration of Fe^{2+} ions that had formed upon irradiation. The Fe^{2+} ion concentration was plotted versus time and the slope, obtained by linear fitting to the equation y = ax +b using Origin software (Supplementary Fig. 10), equals the rate of formation.

Solutions of (*E*)-**2** in the absence and presence of 1 equiv. of $[NBu_4]^+[(S)\text{-}\mathbf{3}]^-$ were irradiated with the Thorlab model F365F1 high-power LED under identical conditions at concentrations high enough to absorb all incident light (Abs365 ≥ 2). The absorbance increase was monitored over time by UV-vis spectroscopy. The molar absorptivities at λ = 380 nm [i.e. for (*E*)-**2**, ε = 618 M^{-1} cm^{-1}; for (*Z*)-**2**, ε = 3505 M^{-1} cm^{-1}; for (*E*)-**2@3**, ε = 1311 M^{-1} cm^{-1}, for (*Z*)-**2@3**, ε = 5030 M^{-1} cm^{-1}] were used to calculate the concentration increase of (*Z*)-**2** according to Δc = $\Delta abs/\varepsilon_E + \varepsilon_Z$. The initial concentration increase was plotted versus time (Supplementary Figs. 12 and 13) and the slope, representing the rate of formation, was obtained by linear fitting to the equation y = ax +b using Origin software. The photochemical quantum yield was calculated by comparison of the rate of formation of (*Z*)-**2** with the rate of Fe^{2+} ion formation from potassium ferrioxalate using the known quantum yield for the latter process (Φ = 1.21).[4]

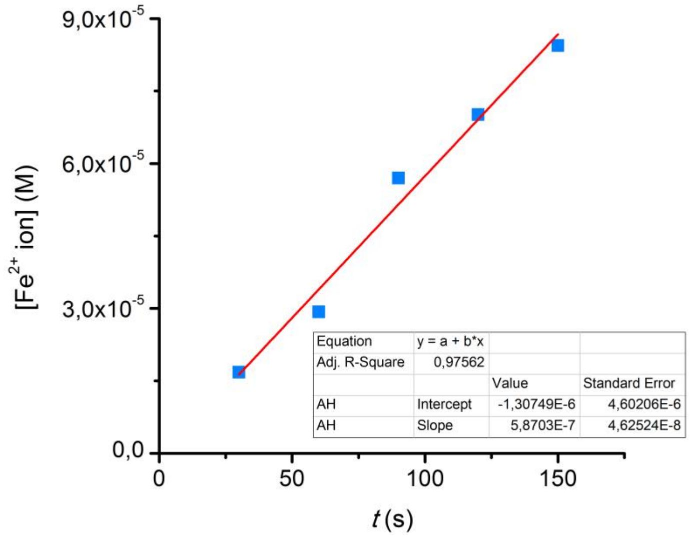

Supplementary Figure 10. Concentration of Fe^{2+} ions measured after five different irradiation times (30, 60, 90, 120, 150 s). The slope of the plot corresponds to the rate of Fe^{2+} ion formation: 5.87×10^{-7} M $s^{-1} \pm 4.62 \times 10^{-8}$ M s^{-1}.

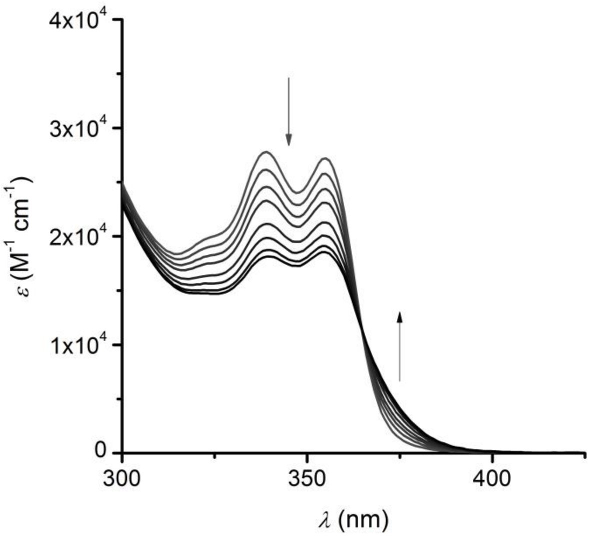

Supplementary Figure 11. UV-vis spectral changes upon irradiation of a solution of (*E*)-**2** in CH_2Cl_2 ($c = 2.0 \times 10^{-4}$ M, 1 mm quartz cuvette) with λ_{max} = 365 nm light.

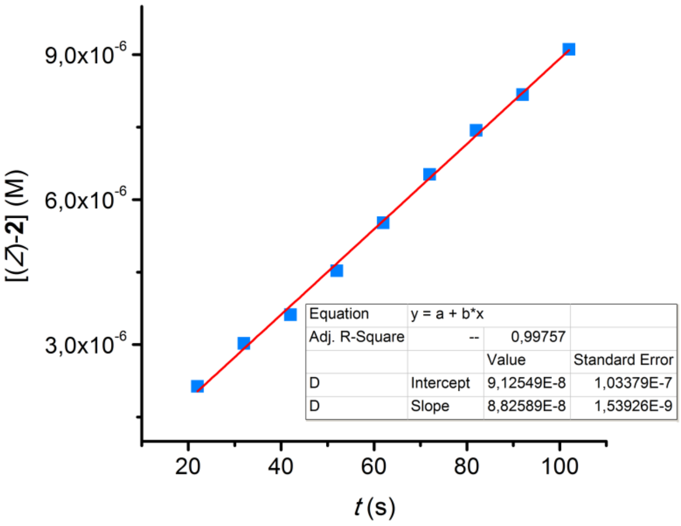

Supplementary Figure 12. Plot of the concentration of (Z)-**2** as a function of time during λ_{max} = 365 nm irradiation of a solution of (E)-**2** in CH_2Cl_2 (c = 3.0 × 10^{-4} M, 1 cm quartz cuvette) obtained by monitoring the absorption increase at λ = 380 nm. The slope of the plot corresponds to the $E \rightarrow Z$ isomerisation rate: 8.83 × 10^{-8} M s^{-1} ± 1.54 × 10^{-9} M s^{-1}.

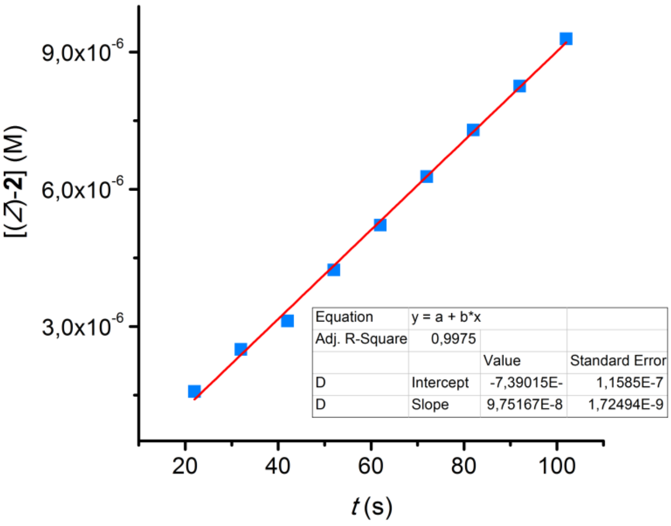

Supplementary Figure 13. Plot of the concentration of (Z)-**2** as a function of time during λ_{max} = 365 nm irradiation of a mixture of (E)-**2** and $[NBu_4]^+$[(S)-**3**]$^-$ in CH_2Cl_2 (c = 2.0 × 10^{-4} M, 1 cm quartz cuvette) obtained by monitoring the absorption increase at λ = 380 nm. The slope of the plot corresponds to the $E \rightarrow Z$ isomerisation rate: 9.75 × 10^{-8} M s^{-1} ± 1.72 × 10^{-9} M s^{-1}.

^{1}H, ^{13}C and ^{31}P NMR Spectra

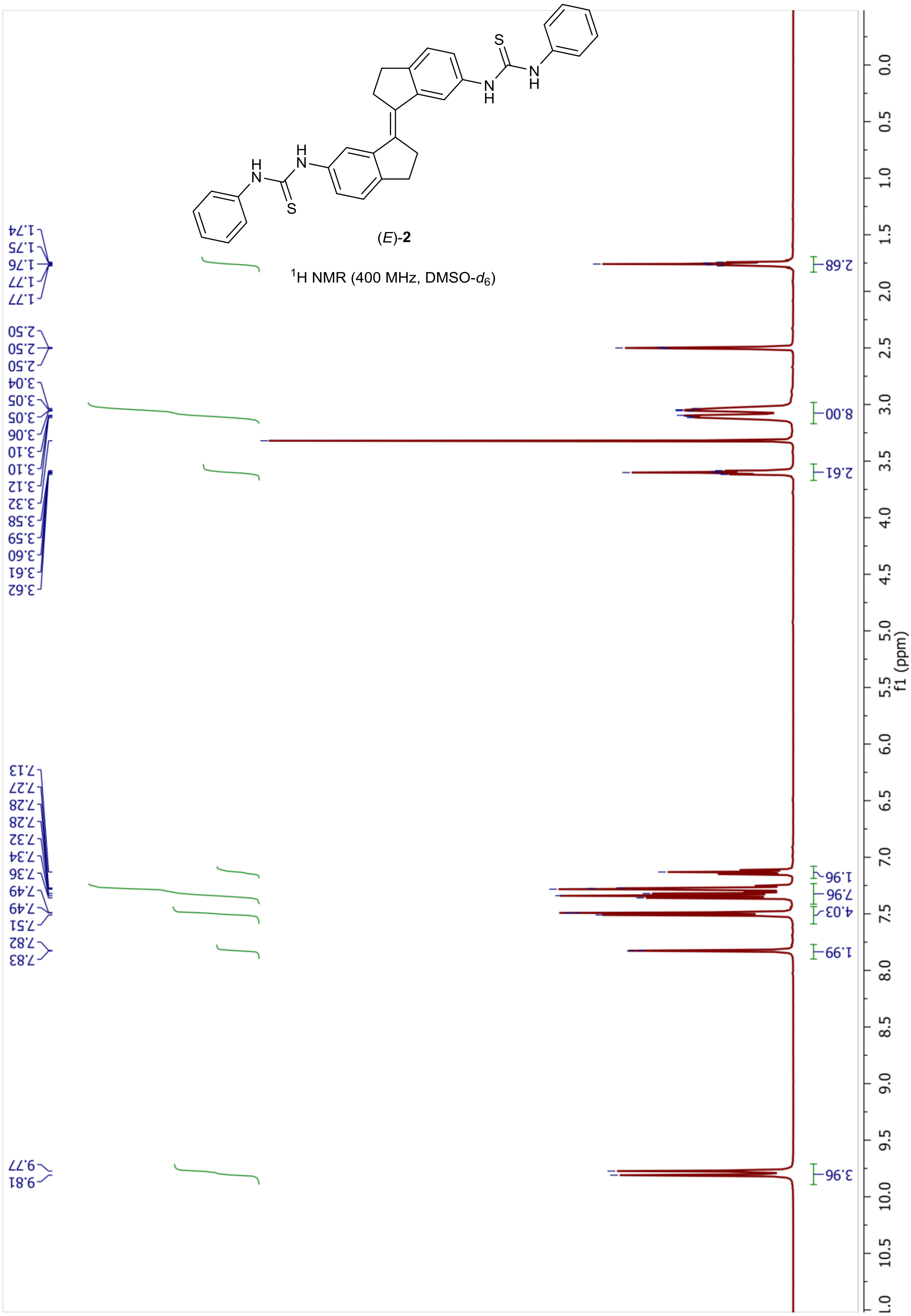

Supplementary Figure 14. ^{1}H NMR spectrum (400 MHz, DMSO-d_6) of compound (*E*)-**2**.

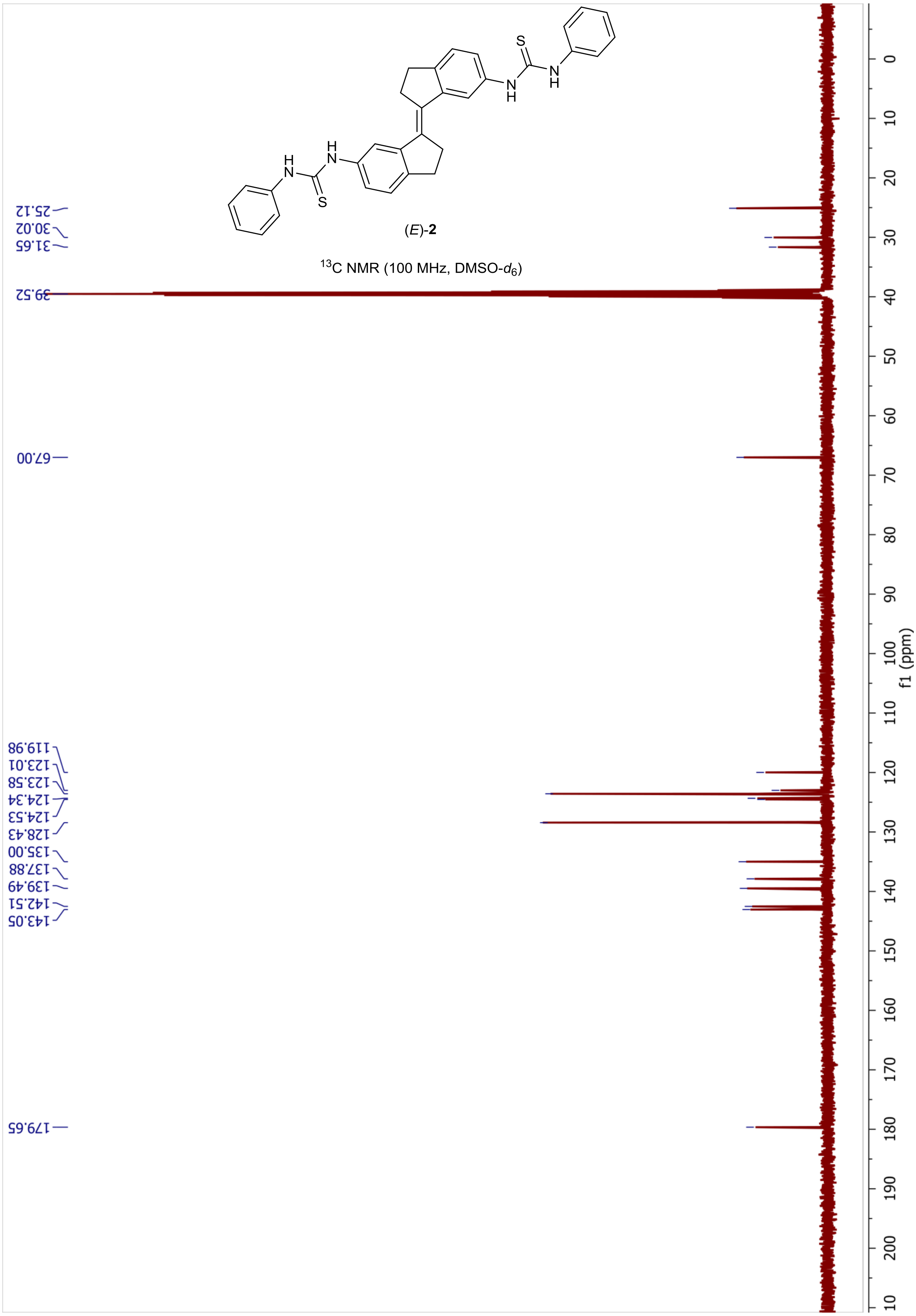

Supplementary Figure 15. ^{13}C NMR spectrum (100 MHz, DMSO-d_6) of compound (*E*)-**2**.

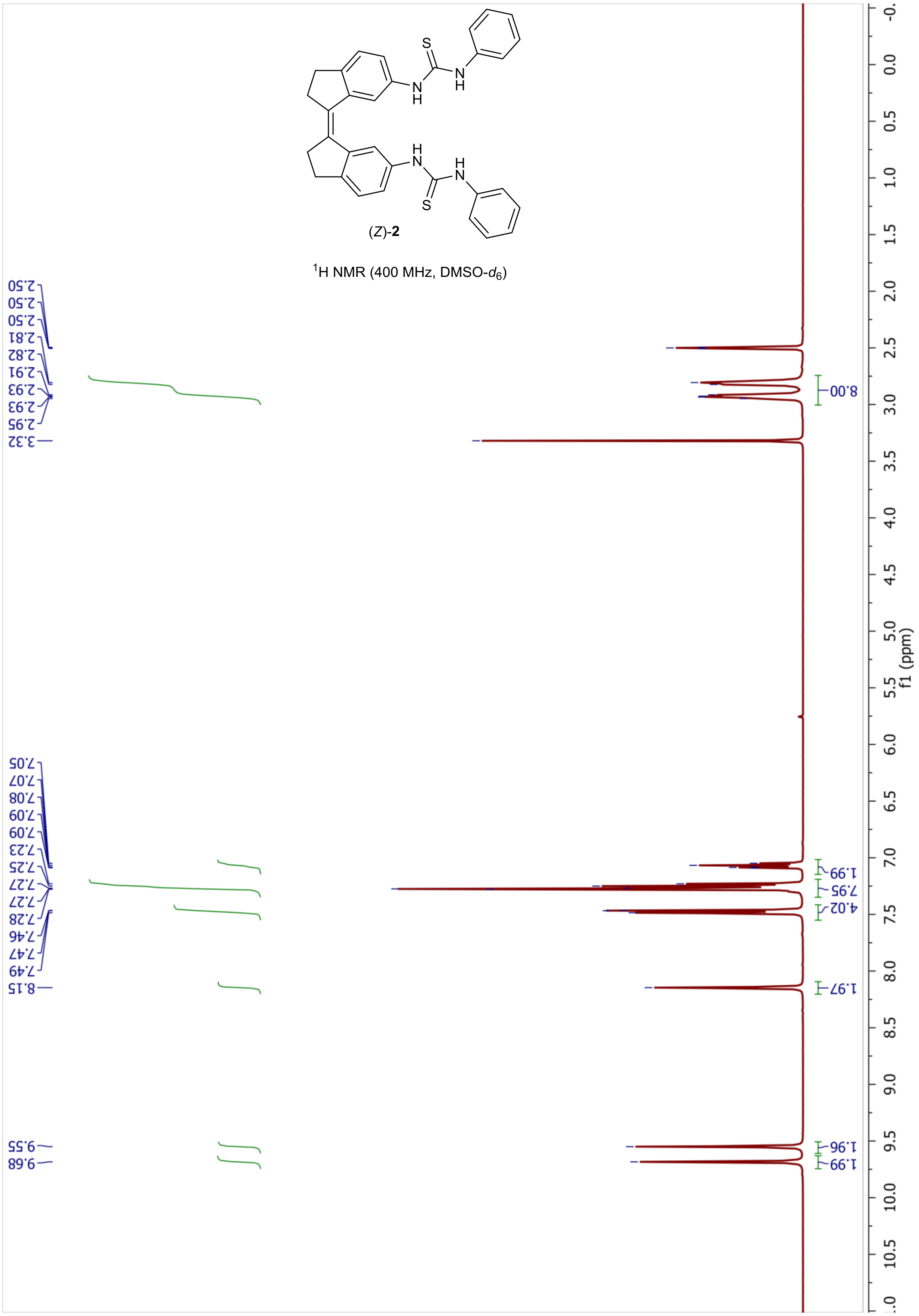

Supplementary Figure 16. ^{1}H NMR spectrum (400 MHz, DMSO-d_6) of compound (Z)-**2**.

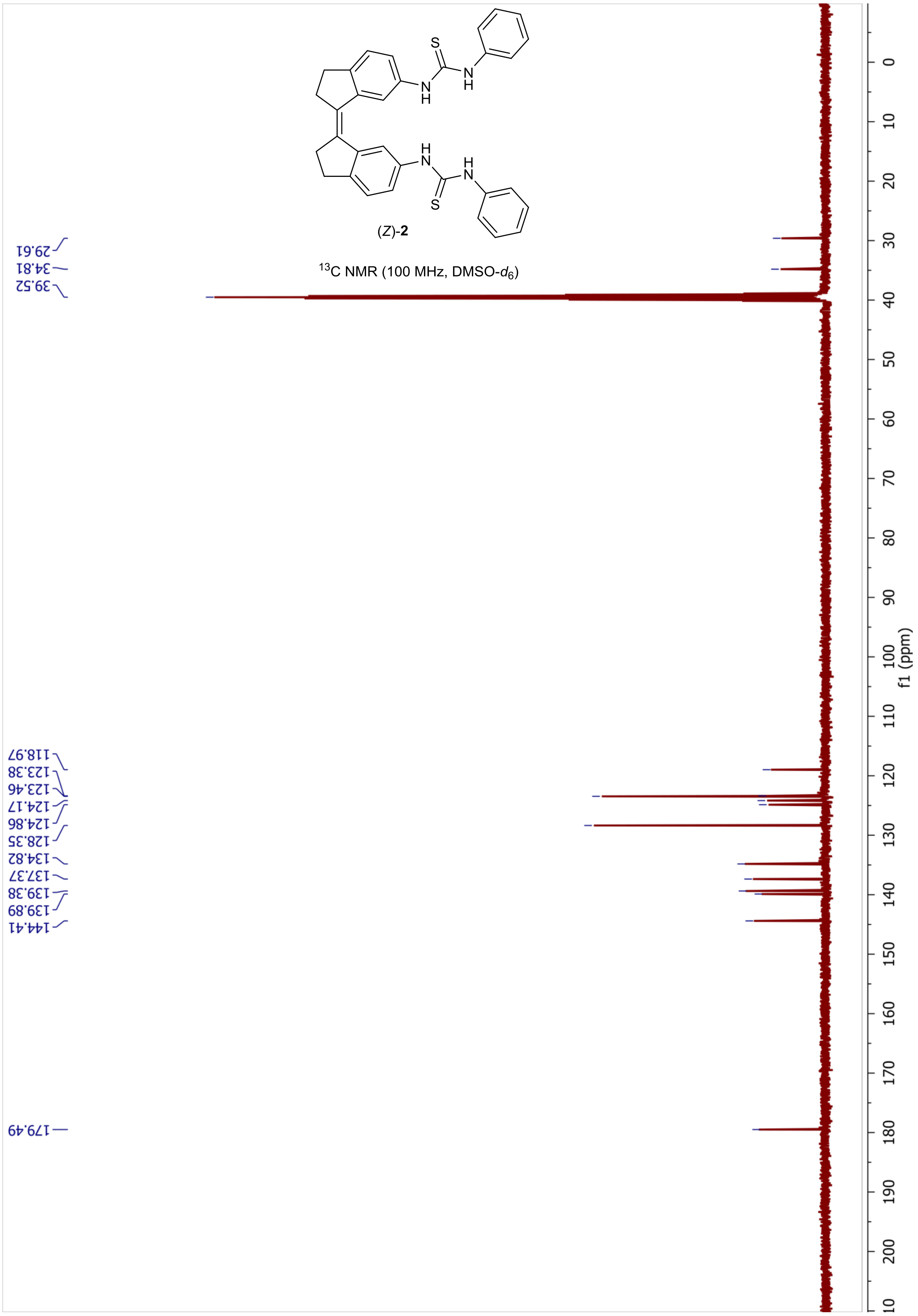

Supplementary Figure 17. ^{13}C NMR spectrum (100 MHz, DMSO-d_6) of compound (*Z*)-**2**.

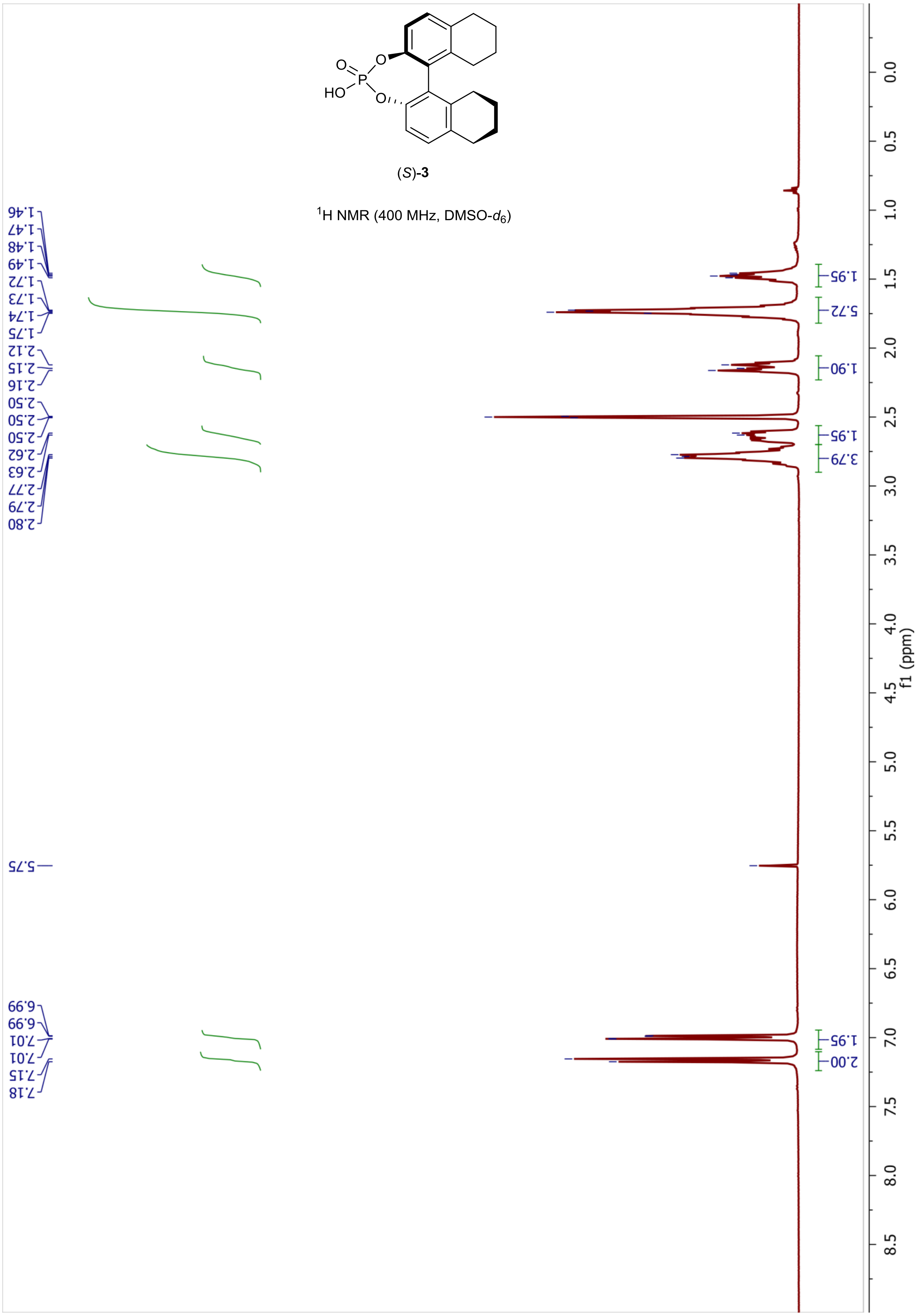

Supplementary Figure 18. ^{1}H NMR spectrum (400 MHz, DMSO-d_6) of compound (S)-**3**.

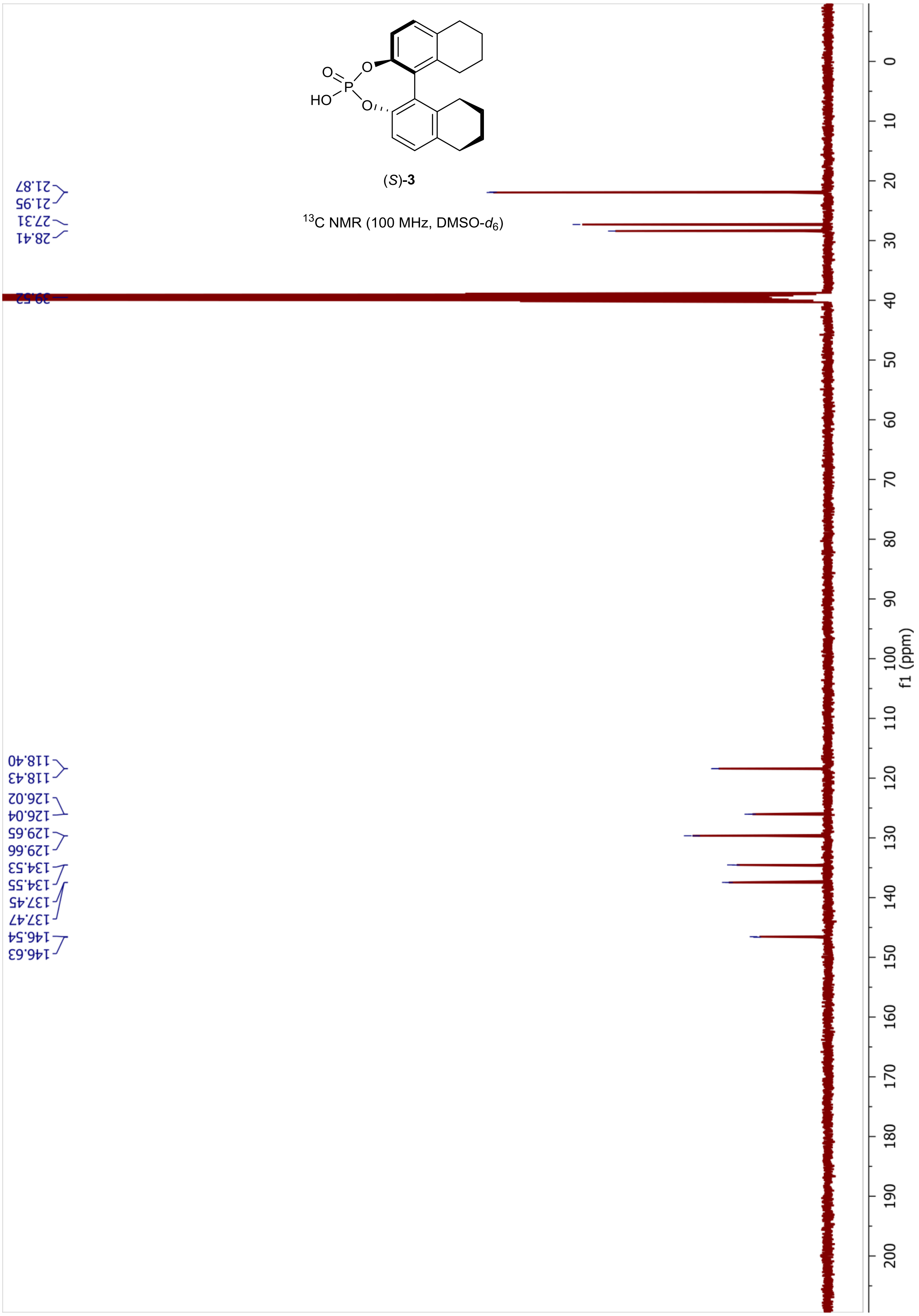

Supplementary Figure 19. ^{13}C NMR spectrum (100 MHz, DMSO-d_6) of compound (*S*)-**3**.

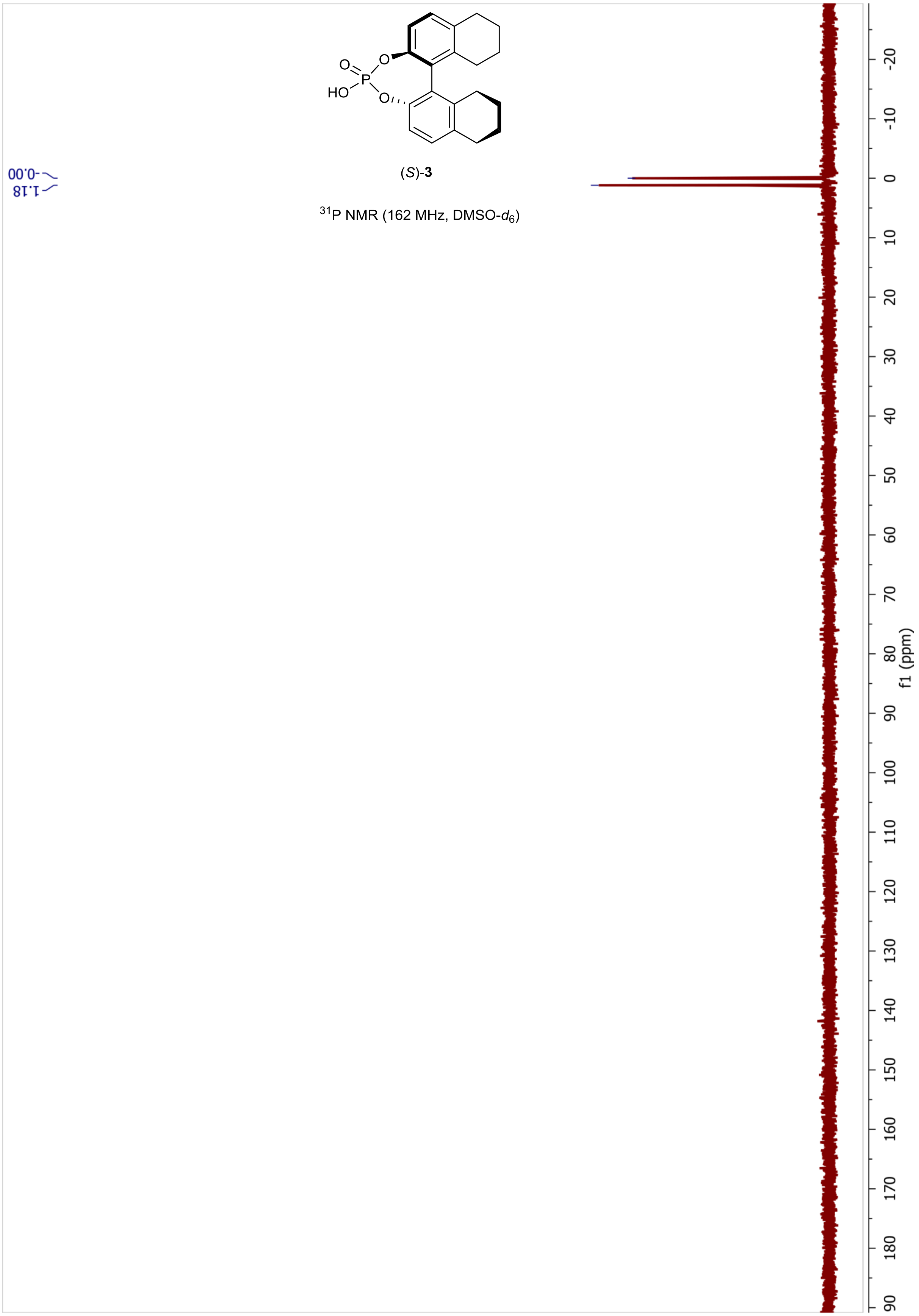

Supplementary Figure 20. ^{31}P NMR spectrum (162 MHz, DMSO-d_6) of compound (*S*)-**3**.

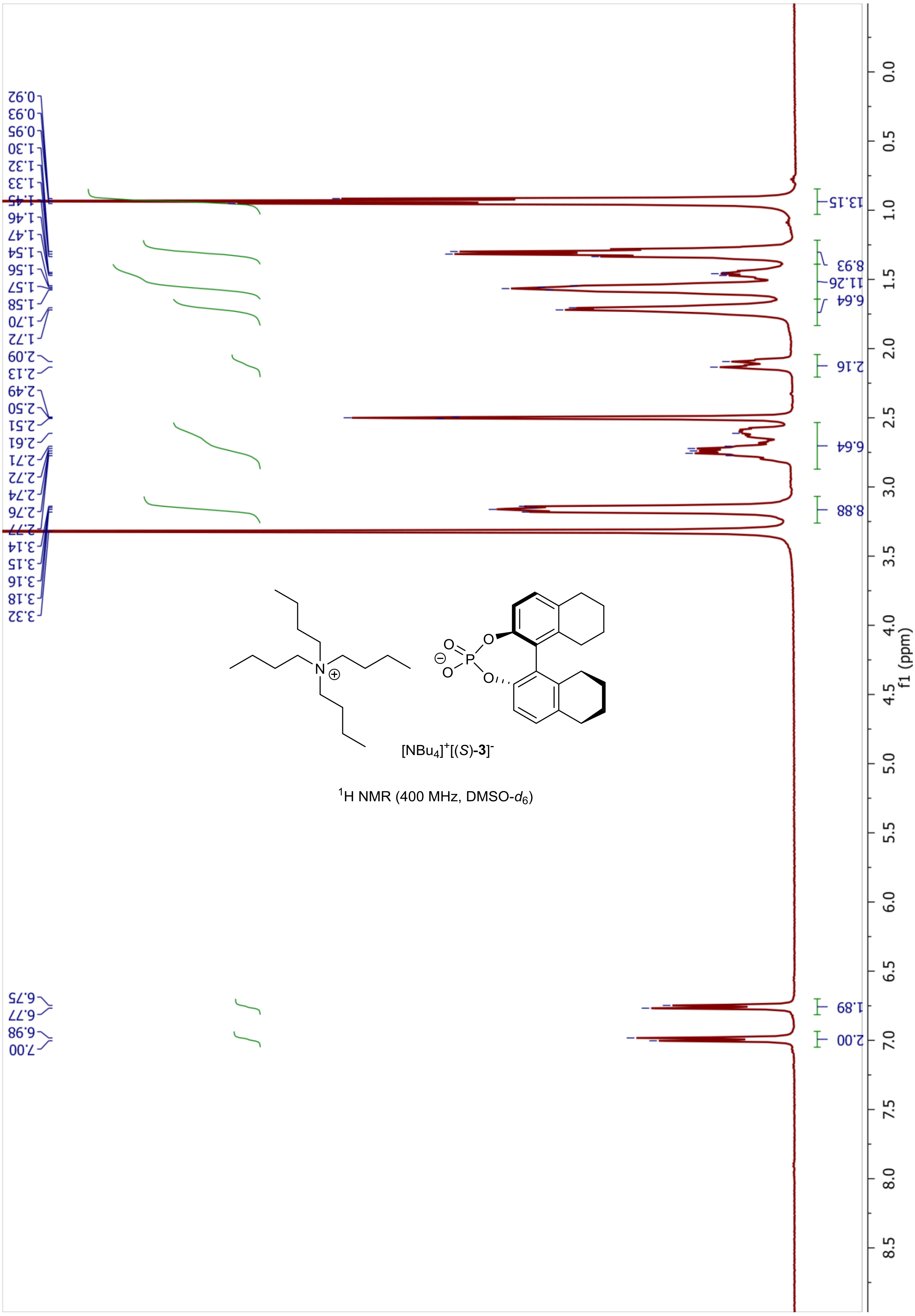

Supplementary Figure 21. ^{1}H NMR spectrum (400 MHz, DMSO-d_6) of $[NBu_4]^+[(S)\text{-}\mathbf{3}]^-$.

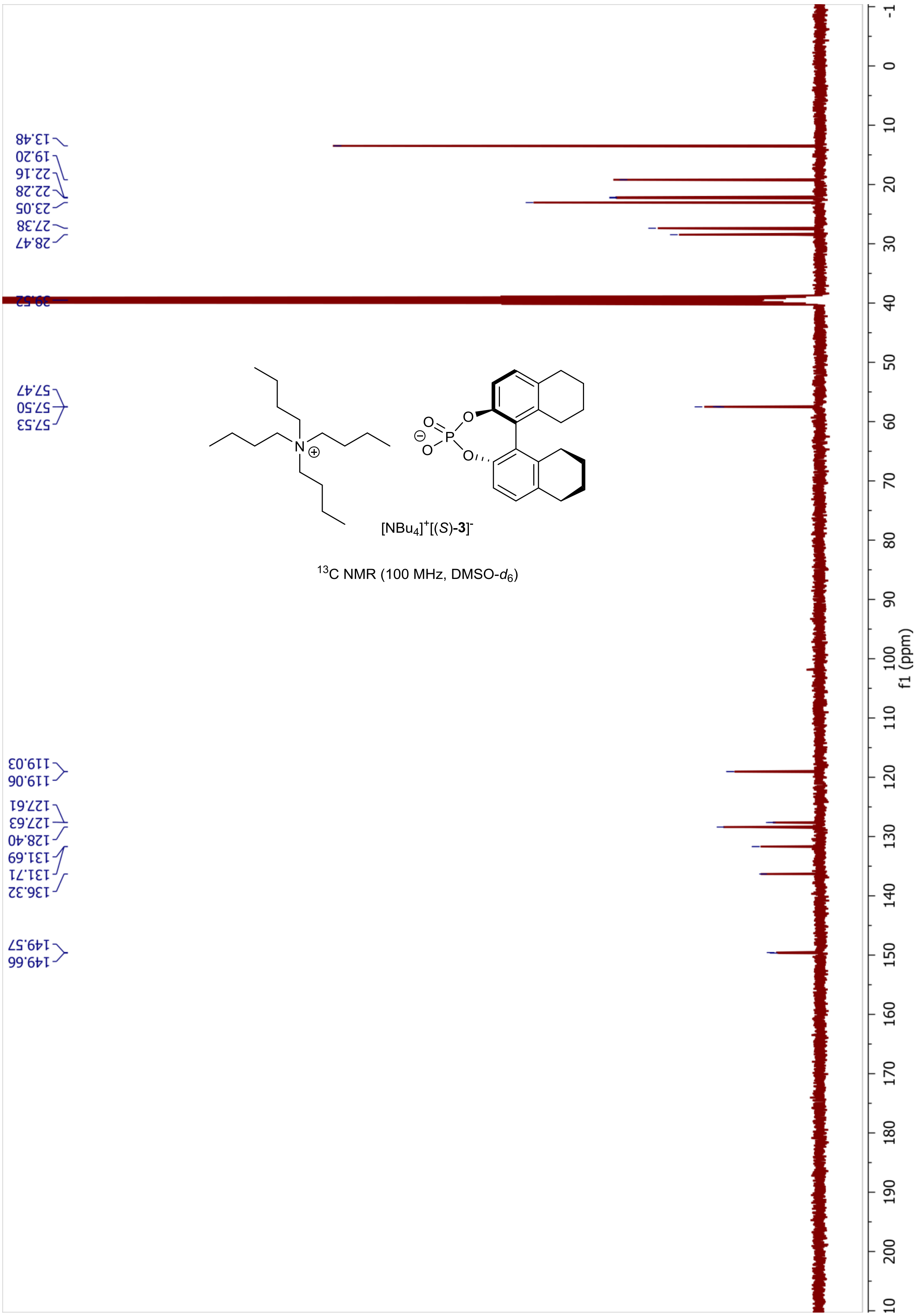

Supplementary Figure 22. ^{13}C NMR spectrum (100 MHz, DMSO-d_6) of [NBu$_4$]$^+$[(*S*)-**3**]$^-$.

Supplementary References

1. Wezenberg, S. J. & Feringa, B. L. Photocontrol of anion binding affinity to a bis-urea receptor derived from stiff-stilbene. *Org. Lett.* **19**, 324–327 (2017).
2. Gaussian 09, Revision D.01, Frisch, M. J., et al. Gaussian, Inc., Wallingford CT (2013).
3. González, A. S., Benitez, D., Tkatchouk, E., Goddard III, W. A. & Toste, F. D. Phosphoramidite gold(I)-catalyzed diastereo- and enantioselective synthesis of 3,4-substituted pyrrolidines. *J. Am. Chem. Soc.* **133**, 5500–5507 (2011).
4. Hatchard, C. G. & Parker, C. A. A new sensitive chemical actinometer. II. Potassium ferrioxalate as a standard chemical actinometer. *Proc. Roy. Soc.* **A235**, 518–536 (1956).
